# The challenge of measuring multi-morbidity and its costs

**DOI:** 10.1186/2045-4015-4-1

**Published:** 2015-01-26

**Authors:** Raphael Wittenberg

**Affiliations:** Personal Social Services Research Unit, London School of Economics and Political Science, Houghton Street, London, WC2A 2AE UK

## Abstract

The ageing of the population across developed countries and beyond has increased the importance of examining multi-morbidity. The recent paper by Arbelle et al. [Isr J of Health Policy Res. 2014;3:29] on multiple chronic conditions in Israel’s Maccabi Health Care System (MHC) is a welcome and interesting contribution to the literature on this topic. They found that the prevalence of multiple chronic conditions among the MHC population rises with age, is lower for higher socioeconomic groups, and is higher than in a primary care population in Scotland studied by Barnett et al. [Lancet. 2012;380:37–43].

The difference in prevalence between the two studies is unlikely to reflect entirely, or probably even mainly, real differences in morbidity rates between the two countries. Systematic reviews have highlighted large differences in the prevalence of multi-morbidity in different studies. Although the Israeli and Scottish study used similar definitions and methods, the nature of the source data differed. It seems likely that the incentives to record the full range of patients’ conditions may differ between data sources depending on the uses of the data, which may in turn depend on the country’s health care financing system. If this is correct, it will complicate comparisons between different jurisdictions.

It is important to consider not only the prevalence of multi-morbidity but also its costs to the health system and to wider society. Cost of illness studies can be helpful in informing decisions about prioritisation of resources. Multi-morbidity complicates such studies. The overall costs of health and social care for people with a specific condition would include costs relating to any comorbidities. To examine the marginal impact on overall costs of each condition among those with multiple conditions is likely to be complex and arguably not especially useful.

## Main text

The ageing of the population across developed countries and beyond has increased the importance of examining multi-morbidity. The recent paper by Arbelle et al. [1] on multiple chronic conditions in Israel’s Maccabi Health Care System (MHC) is a welcome and interesting contribution to the literature on this topic. The authors show that the prevalence of two or more chronic conditions among the two million members of the Maccabi Healthcare Service is 38% and that it rises sharply with age reaching over 90% after age 75 years.

The high prevalence of multi-morbidity clearly presents challenges for health care systems. As patients live longer lives, they are at rising risk of developing multiple chronic disorders. The management of combinations of disorders is likely to be more complex than the management of single conditions. Arbelle et al. conclude that ‘to effectively deal with multiple chronic conditions health care systems must devise strategies, including but not limited to, information technologies that enable shared teamwork based on clinical guidelines which address the problem of multiple, as opposed to single chronic disorders in patients’ [1].

The authors used a similar methodology to that used in a recent Scottish study of multi-morbidity by Barnett et al. [2]. They found that the prevalence of multiple chronic conditions among the MHC population is Israel is statistically significantly higher than the prevalence in the Scottish study population. The prevalence in the Scottish population of two or more chronic conditions was 23.2% as against 38.1% in the MHC population. This is despite differences in the age profile of the two populations: Scotland has a higher proportion of older people and lower proportion of children and young people than Israel.

The difference in prevalence between the two studies is unlikely to reflect entirely, or probably even mainly, real differences in morbidity rates between the two countries. A systematic review of the literature by Marengoni et al. [3] found that the prevalence of multi-morbidity, defined as prevalence of two or more concurrent diseases, varied widely between studies, in the case of older people from 55% to 98%. Fortin et al. [4] also found in their systematic review of prevalence studies of multi-morbidity marked variation in prevalence rates between different studies especially at age 75, where prevalence in the general population varied between 13.1% and 71.8%. The studies used different definitions of multi-morbidity and different recruitment methods. Fortin et al. recommend that ‘investigators should carefully consider the specific diagnoses included and their number, as well as the operational definition of multimorbidity’ [4].

Arbelle et al. [1] did use the same definition and general approach as Barnett et al. [2]. What differed, apart from the country in which the study was conducted, is the nature of the source of the data. While the Israeli study used data from an agency which acts as insurer, care manager and care provider, the Scottish study used data from primary care records. The Israeli MHC data on chronic conditions is used as an input to care management and can be accessed by all physicians working within the MHC plan. The Scottish data derive from the records of the general practices with which the patients are registered.

It is possible that use of different types of data sources yields different prevalence rates of multiple chronic conditions. It seems likely in particular that the incentives to record the full range of patients’ conditions may differ between data sources depending on whether the data source is used for any finance purposes. Where it is used for financial purposes, much may depend on the nature of the country’s health care financing system. If this is correct, it will complicate comparisons between different jurisdictions.

The choice of the appropriate method and data source for estimating the prevalence of multiple chronic conditions should take account of the purposes for which the prevalence estimates are to be used. This raises various questions about the underlying objective of estimating the prevalence of multi-morbidity. It is important to be clear which types of decisions on policy and practice such information is intended to inform. It may be important to estimate not just the prevalence of multi-morbidity in general but to identify common combinations of conditions where the specific combination of conditions requires differences in treatment from the individual conditions. It may also be important to understand not just the overall prevalence of multi-morbidity but also how prevalence varies by age, gender, socioeconomic group and other population characteristics.

Arbelle et al. [1] found that the prevalence of multiple chronic conditions rises sharply with age. This means that unless there is a significant compression of morbidity demand for long term care will rise over the coming decades as the population ages. As the authors stress, there will be ‘an increasing need for tertiary as well as secondary and primary prevention to prevent poor outcomes of combinations of chronic disorders’ [1]. This indicates the importance of developing effective ways to prevent chronic conditions which tend to be associated with other conditions in combinations which may be complex and costly to manage.

The steep age gradient also means that unless there is as high a quality of health and social care for people with multiple chronic conditions as for people with a single condition there is a risk that people in late old age will receive lower quality care than younger people who are less likely to have multiple conditions. Age differences in quality of care, and access to some forms of care, can be a cause for concern.

The study by Arbelle et al. found that, except for children and adolescents, the prevalence of multiple chronic conditions is higher among lower socioeconomic groups. Marengoni et al. [3] found in their systematic review of the literature that factors associated with multi-morbidity include low socioeconomic status as well as older age and female gender. Charlton et al. [5] specifically investigated the impact of deprivation on the occurrence, outcomes and health care costs of people with multiple morbidity in England. They found that the higher incidence of disease, associated with deprivation, channels deprived populations into categories of multiple morbidity with a greater prevalence of depression, higher mortality and higher costs. They conclude that this has implications for the way resources are allocated between areas in England’s NHS. More generally improved care for people with multiple chronic conditions seems important in the context of reducing health inequalities.

It is important to consider not only the prevalence of multi-morbidity but also its costs to the health system and to wider society. Lehnert et al. [6] conducted a systematic review of literature on health care utilisation and costs of older people with multiple chronic conditions. Studies they reviewed found that elders with more chronic conditions had consistently more physician visits, experienced more hospital stays and inpatient bed days, and consumed more pharmaceuticals. Several of the studies they reviewed showed a ‘curvilinear, nearly exponential relationship’ in which costs roughly doubled for each additional chronic condition. This finding suggests that the costs associated with individual conditions cannot simply be summed to produce an estimate of the costs of combinations of conditions. They comment that ‘little is known about the natural clustering of diseases, little more about the prevalence of specific disease combinations’ and recommend that future studies investigating health care costs should take chronic conditions into account.

Austerity following the recent financial crisis has increased the importance of ensuring maximum health gain from limited resources. This has increased the value of cost of illness studies, which examine the economic impact of specific health conditions on the public finances, the health and care system and society more widely.

The relative costs of different health conditions is one of the factors that should be taken into account in considering priorities for service development and for research. Such studies tend to examine the full costs of health and social care for people with the condition under consideration. To examine the marginal costs due to one condition when people have with several conditions would be complex and probably liable to a considerable degree of uncertainty. Prince et al. [7] in their recent work on the costs of dementia in the United Kingdom (UK), for example, estimated the costs of all health and social care for people with dementia and not just the costs of that part of their care that relates to dementia.

People with dementia frequently have comorbidities, for example the proportion of people with dementia who also have diabetes is higher than the proportion of people of the same age without dementia who have diabetes. To try to split the costs of care for people with dementia between costs of care for dementia, costs of care for diabetes and costs of care for other comorbidities would be complex. It is also questionable whether it would be useful. It is important to recognise however that the sum of the costs of health and social care summed across studies of the costs of different conditions involve double-counting of some services and that if a specific health condition could be prevented only part of the costs estimated for it in cost of illness studies would be saved.

There have been calls for improved care for people with multiple chronic conditions. Tinetti et al. [8] point out that ‘the most common chronic condition experienced by adults is multimorbidity’ and argue that to ensure safe and effective care for adults with multiple chronic conditions ‘health care must shift its current focus on managing innumerable individual diseases’ to a focus on managing multiple conditions. Vogeli et al. [9] conclude that ‘understanding how to care effectively for persons with multiple chronic conditions is among the most important challenges our health care system faces’. Arbelle et al. [1] stress the need for clinical guidelines to be improved and adapted to account for patients with multiple chronic conditions.

There have also been calls for more research into multiple chronic conditions. Tinetti et al. [6], for example, comment that research funding organisations and industry must ensure that research generates evidence that accurately informs decision making for patients with multiple chronic conditions. In a welcome recent development the English National Institute for Health Research (NIHR) has recently issued a call for applications for research into the evaluation of interventions or services delivered for older people with multimorbidity [10].

Commentary on Arbelle J E, Chodick G, Goldstein A and Porath P (2014) Multiple chronic disorders - health care system’s modern challenge in the Maccabi Health Care System, *Israel Journal of Health Policy Research.*

## Author information

Raphael Wittenberg is a Principal Research Fellow at the Personal Social Services Research Unit, London School of Economics and Political Science, and Deputy Director of the Centre for Health Service Economics and Organisation, University of Oxford, with a special interest in the financing of long-term care and the economics of services for older people.

## Commentary on

Arbelle J E, Chodick G, Goldstein A and Porath P. Multiple chronic disorders - health care system’s modern challenge in the Maccabi Health Care System
